# Subjective life expectancy is a risk factor for perceived health status and mortality

**DOI:** 10.1186/s12955-017-0763-0

**Published:** 2017-10-02

**Authors:** Jae-Hyun Kim, Jang-Mook Kim

**Affiliations:** 1 Department of Health Administration, College of Health Science, Dankook University, 201, Manghyang-ro, Dongnam-gu, Cheonan-si, 330-714 Chungnam Republic of Korea; 2 Institute of Health Promotion and Policy, Dankook University, Cheonan, Republic of Korea

**Keywords:** Expectancy, Mortality, Life, Health

## Abstract

**Background:**

The purpose of this study was to investigate the association between subjective life expectancy (SLE) and self-rated health and further SLE will predict higher risk for mortality.

**Methods:**

Data from the Korean Longitudinal Study of Aging (KLoSA) from 2006 to 2014 was assessed using longitudinal data analysis and 10,244 research subjects were included at baseline in 2006. Our modeling approach was based on generalized estimating equation (GEE) for self-rated health and Cox proportional hazards models for mortality.

**Results:**

SLE was significantly associated with mortality (*p* for trend <0.0001), with the following ORs predicting mortality (yes vs. no): HR = 2.133 (*p* < .0001) for 0%, HR = 1.805 (*p* < .0001) for 10-20%, HR = 1.494 (*p* 0.002) for 30-40%, HR = 1.423 (*p* 0.002) for 50-60%, HR = 1.157 (*p* 0.235) for 70-80%, vs. 90-100%. In terms of age-specific association with SLE for self-rated health and mortality, as subjects got older, self-rated health tended to lean more toward poor self-rated health, but as for mortality, the probability of dying increased for people who are younger and HR also tended to increase.

**Conclusion:**

This study has shown that SLE is associated with self-rated health and further is a powerful predictor of mortality after adjusting for self-rated health as well as sociodemographic factors and health risk status and behavior factors in a representative population of Koreans.

## Background

There is a growing interest in subjective measures of health and survival. People have expectations about their remaining length of life, and these expectations appear to make sense [1]. Economic theories often assume that people have expectations that accurately account for the information available. As people get older, the future becomes more meaningful. For example, people in their late-midlife period tended to show significant changes in behavior in economic choices [2], plans [3], predicting mortality [4] and investing in future health (e.g., participating in cancer screening more) [5, 6].

Subjective life expectancy (SLE) indicates an individual’s subjective length of life expectancy, and it provides a personalized timeframe that can act as a guide for apportioning work, leisure, and finances [7]. This subjective measure can sometimes offer information that other objective measures are unable to [8]. Therefore, SLE is being used more to elucidate people’s decisions across various life domains such as health.

Currently, SLE is asked in a number of longitudinal surveys of older persons, such as The Health and Retirement Study (HRS) [8–10], the English Longitudinal Study of Ageing, the Study of Health and Ageing in Europe, and the China Health and Retirement Longitudinal Study. Responses to this question can be predictive measures for mortality even in the presence of other mortality-related characteristics [1, 4, 8]. Furthermore, self-rated health [11] as well as subjective life expectancy (SLE) is predictive of actual mortality. It is considered an inclusive measure of health, meaning that self-rated health yields information inaccessible by targeted health measurements [12]. To support this, negative health ratings seem to represent pathogenetic biological processes in the body that compromise health status and may herald future health adversities [12]. However, the study of Siegel M. et al. [1] shows that although both self-rated health and SLE may be conceptually related, they have independent empirical effect on mortality despite their ability to predict future risk of objective health outcomes such as mortality.

Therefore, we address the following research questions. (1) Does SLE predict poor self-rated health? If so, (2) is SLE an independent predictor of mortality after adjusting for self-rated health? Thus, this study performed two analyses for self-rated health and mortality, respectively. In the first analysis, we estimated the influence of SLE on self-rated health with generalized estimating equation (GEE) model, while adjusting for age, gender, residential region, education, smoking status, alcohol use, labor and number of chronic disease. In second analysis, we estimate the independent effect of SLE as a predictor of mortality using cox proportional hazard model while adjusting for age, gender, residential region, education, smoking status, alcohol use, labor, number of chronic disease and self-rated health.

## Methods

### Study sample & design

Data were obtained from the 2006, 2008, 2010, 2012 and 2014 waves of the Korean Longitudinal Study of Aging (KLoSA). KLoSA conducted a multistage stratified cluster sampling based on 15 geographical areas and housing types across the nation to create nationally representative longitudinal data of Koreans aged 45 years or more by the Korea Labor Institute. In the first baseline survey in 2006, 10,254 individuals in 6171 households (1.7 per household) were interviewed using the Computer-Assisted Personal Interviewing method. There were 292 individuals with cancer. The second survey, in 2008, followed up with 8688 subjects, who represented 86.6% of the original panel. The third survey, in 2010, followed up with 7920 subjects, who represented 80.3% of the original panel, the fourth survey, in 2012, followed up with 7486 subjects, who represented 76.2% of the original panel and the fifth survey, in 2014, followed up with 7029 subjects, who represented 72.8% of the original panel. To estimate the association between SLE and self-rated health and mortality among people 45 years or older, we included 10,244 participants at baseline 2006 with no missing information.

### Independent variables

#### Subjective life expectancy (SLE)

SLE measures a continuum of subjective probabilities by asking “What is the percent chance that you will live to be [75 (if age is 64 or less) / 80 (if age is 65–69) / 85 (if age is 70–74) / 90 (if age is 75–79) / 95 (if age is 80–84) / 100 (if age is 85–94)] / 105 (if age is 95–99) / 110 (if age is 100 or more)]?” The target age in expectation is determined by respondents’ current age. The response to the question ranges from 0 to 100, where 0 means that you think there is absolutely no chance, and 100 means that you think the event is absolutely sure to happen.

### Dependent variables

#### Self-rated health

Self-rated health asks respondents to rate their health, often using a 5-point scale: “excellent,” “very good,” “good,” “fair,” and “poor,” with variations of the response scales such as a 5-point scale of “very good,” “good,” “neither good nor bad,” “bad,” and “very bad”. The response “fair” or “poor” indicated “Bad,” and the response “excellent,” “very good,” or “good” indicated “Good,” thus dichotomizing the response.

#### All-cause mortality

All-cause mortality during the time interval from year 2006 to the end of follow-up was the main outcome of the study. Death over a maximum follow-up period of 8 years was determined by death certificates.

### Control variables

Covariates were collected: age (45–54, 55–64, 65-74 and ≥65 years), gender, residential region (metropolitan, urban and rural), education (elementary, middle, high school, and ≥college), cigarette smoking (non-smoker, former smoker and smoker), alcohol consumption (nothing, former drinker and drinker), labor status (yes and no), and comorbidities of hypertension, diabetes, cancer, chronic obstructive pulmonary disease, liver disease, heart disease, cerebrovascular diseases, mental illness and arthritis or rheumatoid arthritis (0, 1, 2 and ≥3).

### Analytical approach and statistics

Chi-square test, log-rank test, generalized estimating equation (GEE) model and Cox proportional hazards models were used to investigate the association between SLE and self-rated health and mortality. GEE was required in order to handle the unbalanced data with correlated outcomes and missing data. This GEE model assumed proper distributions for each individual while taking into account the correlation among individual. In this study, the correlation structure was modeled as an exchangeable correlation structure. Self-rated health (yes/no) was the outcome in GEE models. Covariates of interest from all subjects were added to the model to determine their effects on the probability of reporting poor self-rated health. Further, to examine the impact of SLE on mortality, adjusted hazard ratio (HR) was calculated by cox proportional hazard model. The outcome variable was survival time, which was measured from date of enrollment to death or censoring (up to 8 years). For all analyses, the criterion for statistical significance was *p* ≤ 0.05, two-tailed. All analyses were conducted using the SAS statistical software package, version 9.4 (SAS Institute Inc., Cary, NC, USA).

## Results

### Sample characteristics

Table 1 shows the baseline characteristics of participants. As shown in Table 1, of the 10,244 individuals at baseline 2006, those with bad self-rated health were 3183 participants (31.1%) and about 11.2% of participants (N: 1143) died during the follow-up period. Grouping the SLE responses, 6.4% of participants (655/10,244) estimated their chance of living another 10–15 years as 0%, 5.7% (579/10,244) estimated a 10-20% chance, 10.4% (1063/10,244) estimated a 30-40% chance, 27.2% (2781/10,244) estimated a 50-60% chance, 26.8% (2740/10,244) estimated a 70-80% chance and 23.7% (2426/10,244) estimated a 90-100% chance. Just over half of participants were female (56.5%; 5786/10,244). Nearly one third had high school educational attainment (26.4%; 2708/10,244) and 47.1% had elementary or lower educational attainment (4823/10,244). Age, residential region, smoking status, alcohol use, labor and number of chronic disease are also shown in Table 1.

**Table 1:** General characteristics of participants at baseline

	Total		Perceived health status				*P*-value	Death				P-value
			Bad		Good			No		Yes		
	N	%	N	%	N	%		N	%	N	%	
Subjective life expectancy (point)							<.0001					<.0001
0	655	6.4	451	68.9	204	31.2		444	67.8	211	32.2	
10-20	579	5.7	346	59.8	233	40.2		441	76.2	138	23.8	
30-40	1063	10.4	519	48.8	544	51.2		869	81.8	194	18.3	
50-60	2781	27.2	959	34.5	1822	65.5		2469	88.8	312	11.2	
70-80	2740	26.8	559	20.4	2181	79.6		2560	93.4	180	6.6	
90-100	2426	23.7	349	14.4	2077	85.6		2318	95.6	108	4.5	
Age							<.0001					<.0001
45-54	3293	32.2	406	12.3	2887	87.7		3227	98.0	66	2.0	
55-64	2791	27.3	783	28.1	2008	72.0		2630	94.2	161	5.8	
65-74	2679	26.2	1143	42.7	1536	57.3		2317	86.5	362	13.5	
≥ 74	1481	14.5	851	57.5	630	42.5		927	62.6	554	37.4	
Gender												<.0001
Male	4458	43.5	1043	23.4	3415	76.6		3844	86.2	614	13.8	
Female	5786	56.5	2140	37.0	3646	63.0		5257	90.9	529	9.1	
Residential region							0.001					0.003
Metropolitan	1765	17.2	507	28.7	1258	71.3		1605	90.9	160	9.1	
Urban	2967	29.0	875	29.5	2092	70.5		2645	89.2	322	10.9	
Rural	5512	53.8	1801	32.7	3711	67.3		4851	88.0	661	12.0	
Education							<.0001					<.0001
≤ Elementary	4823	47.1	2294	47.6	2529	52.4		4017	83.3	806	16.7	
Middle school	1656	16.2	395	23.9	1261	76.2		1534	92.6	122	7.4	
High school	2708	26.4	374	13.8	2334	86.2		2550	94.2	158	5.8	
≥ College	1057	10.3	120	11.4	937	88.7		1000	94.6	57	5.4	
Smoking status							<.0001					<.0001
Non-smoker	7291	71.2	2413	33.1	4878	66.9		6580	90.3	711	9.8	
Former smoker	978	9.6	318	32.5	660	67.5		801	81.9	177	18.1	
Smoker	1975	19.3	452	22.9	1523	77.1		1720	87.1	255	12.9	
Alcohol consumption							<.0001					<.0001
Nothing	3884	37.9	803	20.7	3081	79.3		3523	90.7	361	9.3	
Former drinker	689	6.7	370	53.7	319	46.3		536	77.8	153	22.2	
Drinker	5671	55.4	2010	35.4	3661	64.6		5042	88.9	629	11.1	
Labor							<.0001					<.0001
Yes	3953	38.6	612	15.5	3341	84.5		3775	95.5	178	4.5	
No	6291	61.4	2571	40.9	3720	59.1		5326	84.7	965	15.3	
Number of chronic diseases^a^							<.0001					<.0001
0	5470	53.4	775	14.2	4695	85.8		5043	92.2	427	7.8	
1	2945	28.8	1140	38.7	1805	61.3		2566	87.1	379	12.9	
2	1265	12.4	789	62.4	476	37.6		1050	83.0	215	17.0	
≥ 3	564	5.5	479	84.9	85	15.1		442	78.4	122	21.6	
Total	10,244	100.0	3183	31.1	7061	68.9		9101	88.8	1143	11.2	

^a^Hypertension, diabetes, cancer, chronic obstructive pulmonary disease, liver disease, heart disease, cerebrovascular diseases, mental illness and arthritis or rheumatoid arthritis

### Relationship between subjective life expectation (SLE) and self-rated health

In the fully adjusted model (Table 2), SLE was associated with self-rated health in a statistically significant linear dose response fashion (*p* for trend <0.0001), with the following ORs predicting self-rated health (yes vs. no): OR = 6.778 (*p* < .0001) for 0%, OR = 3.940 (*p* < .0001) for 10-20%, OR = 2.936 (*p* < .0001) for 30-40%, OR = 2.094 (*p* < .0001) for 50-60%, OR = 1.483 (*p* < .0001) for 70-80%, vs. 90-100% (Table 2). Older age was positively associated with self-rated health (OR = 2.415; *p* < .0001), as was having elementary or lower educational attainment (OR = 2.974; *p* < .0001 vs. college or more educational attainment), being a current smoker (OR = 1.140; *p* < .0006), being alcohol drinker (OR = 2.193; *p* < .0001) and having complex comorbidity (OR = 11.541; *p* < .0001; Table 2).

**Table 2:** Adjusted effect of subjective life expectancy on perceived health status and death

	Self-rated health			Death		
	OR	SE	P-value	HR	SE	P-value
Subjective life expectancy (%)						
0	6.778	0.069	<.0001	2.130	0.131	<.0001
10-20	3.940	0.060	<.0001	1.805	0.137	<.0001
30-40	2.936	0.055	<.0001	1.494	0.128	0.002
50-60	2.094	0.049	<.0001	1.423	0.116	0.002
70-80	1.483	0.051	<.0001	1.157	0.123	0.235
90-100	1.000			1.000		
Age						
45-54	1.000			1.000		
55-64	1.393	0.045	<.0001	2.138	0.151	<.0001
65-74	1.959	0.046	<.0001	3.850	0.146	<.0001
≥ 74	2.415	0.051	<.0001	10.193	0.150	<.0001
Gender						
Male	0.693	0.038	<.0001	2.164	0.082	<.0001
Female	1.000			1.000		
Residential region						
Metropolitan	1.000			1.000		
Urban	0.944	0.040	0.150	1.428	0.098	0.000
Rural	1.098	0.036	0.010	1.420	0.091	0.000
Education						
≤ Elementary	2.974	0.057	<.0001	1.458	0.145	0.009
Middle school	1.797	0.061	<.0001	1.250	0.162	0.170
High school	1.246	0.060	0.000	1.213	0.155	0.213
≥ College	1.000			1.000		
Smoking status						
Non-smoker	1.000			1.000		
Former smoker	0.780	0.044	<.0001	0.703	0.087	<.0001
Smoker	1.140	0.048	0.006	0.912	0.100	0.360
Alcohol use						
Nothing	1.000			1.000		
Former drinker	0.924	0.033	0.016	0.898	0.080	0.179
Drinker	2.196	0.046	<.0001	1.011	0.103	0.918
Labor						
Yes	1.000			1.000		
No	1.781	0.030	<.0001	1.723	0.092	<.0001
Number of chronic diseases^a^						
0	1.000			1.000		
1	1.705	0.045	<.0001	0.986	0.073	0.844
2	3.975	0.066	<.0001	1.088	0.089	0.343
≥ 3	11.541	0.127	<.0001	1.187	0.111	0.121
Self-rated health						
Good				1.000		
Bad				1.575	0.070	<.0001

^a^Hypertension, diabetes, cancer, chronic obstructive pulmonary disease, liver disease, heart disease, cerebrovascular diseases, mental illness and arthritis or rheumatoid arthritis

### Relationship between subjective life expectation (SLE) and mortality

**Fig. 1:**
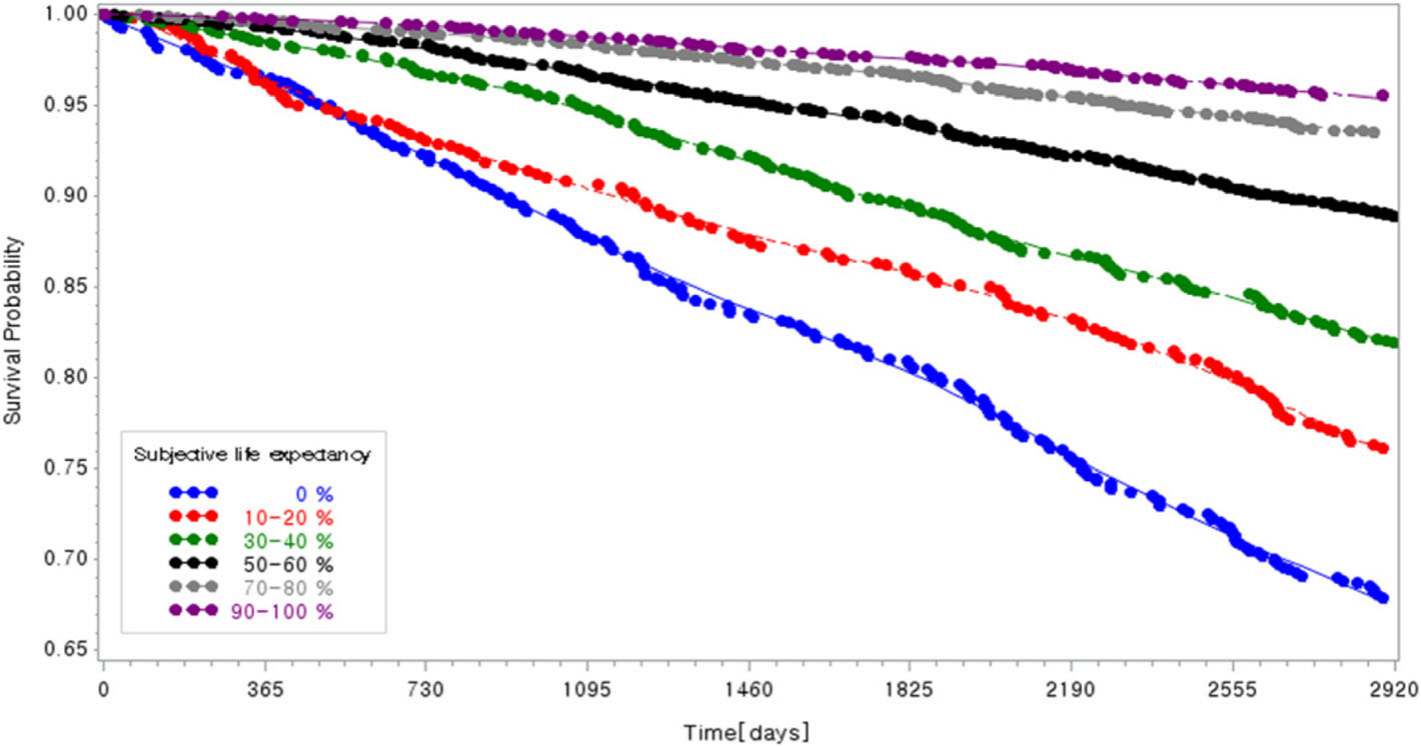
Kaplan-Meier curve for mortality

Figure 1 shows the Kaplan-Meier curve for all-cause mortality. All of log-rank test were statistically significant. In the fully adjusted model including self-rated health (Table 2), SLE was also associated with mortality in a statistically significant linear dose response fashion (*p* for trend <0.0001), with the following ORs predicting mortality (yes vs. no): HR = 2.133 (*p* < .0001) for 0%, HR = 1.805 (*p* < .0001) for 10-20%, HR = 1.494 (*p* 0.002) for 30-40%, HR = 1.423 (*p* 0.002) for 50-60%, HR = 1.157 (*p* 0.235) for 70-80%, vs. 90-100% (Table 3). Older age was positively associated with mortality (HR = 10.193; *p* < .0001), as was having elementary or lower educational attainment (HR = 1.458; *p* 0.009 vs. college or more educational attainment) and having poor self-rated health (HR = 1.575; *p* < .0001; Table 2).

**Table 3:** Age-specific association of subjective life expectancy with self-rated health and death

		Self-rated health^a^			Death^b^		
		OR	SE	P-value	HR	SE	P-value
Age group	Subjective life expectancy (%)						
≤64	0	5.694	0.154	<.0001	2.338	0.331	0.010
	10-20	3.935	0.112	<.0001	1.196	0.379	0.638
	30-40	2.734	0.085	<.0001	2.035	0.252	0.005
	50-60	2.088	0.065	<.0001	1.398	0.201	0.095
	70-80	1.489	0.066	<.0001	1.462	0.189	0.045
	90-100	1.000			1.000		
65-74	0	7.241	0.129	<.0001	1.879	0.232	0.007
	10-20	4.062	0.107	<.0001	1.528	0.236	0.072
	30-40	3.175	0.098	<.0001	1.564	0.206	0.030
	50-60	2.261	0.091	<.0001	1.342	0.184	0.109
	70-80	1.580	0.096	<.0001	0.926	0.204	0.706
	90-100	1.000			1.000		
75-85	0	8.189	0.190	<.0001	1.801	0.274	0.032
	10-20	4.212	0.185	<.0001	1.637	0.281	0.080
	30-40	3.110	0.183	<.0001	1.085	0.278	0.770
	50-60	2.159	0.181	<.0001	1.295	0.270	0.339
	70-80	1.664	0.190	0.007	0.988	0.299	0.968
	90-100	1.000			1.000		

^a^adjusted for gender, residential region, education, smoking status, alcohol use, labor and number of chronic disease

^b^adjusted for gender, residential region, education, smoking status, alcohol use, labor, number of chronic disease and self-rated health

### Age specific relationship between SLE and self-rated health and mortality

Both self-rated health and mortality were positively associated with SLE. The relationship between SLE, self-rated health and mortality was statistically significant (Table 3). As people got older, self-rated health tended to lean more toward poor self-rated health, but as for mortality, the probability of dying increased for people younger and HR also tended to increase.

## Discussion

In this representative cohort study of Korean older people, SLE was a strong positive predictor of self-rated health and mortality over the 8-year study follow-up. Those who rated their chances of living another 10 to 15 years as 0% show the poorest self-rated health and highest mortality risk. This finding was independent of sociodemographic factors and health risk behavior factors for self-rated health and mortality, respectively. It was also independent of self-rated health on mortality risk for older adults, indicating that self-rated health might make unique contributions to predicting risk of mortality.

In this study, strong implications have been observed in regards to SLE influencing economic choice behavior [13, 14], as well as health behavior (e.g., participating in a screening programs) indicating investment for future wellness. [15, 16]. As a result, these behaviors may affect the probability of risk of mortality.

Regarding age-specific association with self-rated heath, as people got older, self-rated health tended to lean more toward poor self-rated health. One potential reason could be the possibility of loss aversion. Van Nooten FE et al. [17] examined whether SLE impacts the willingness to trade-off (WTT) and the number of years traded-off in a 10-year time trade-off (TTO) exercise to assess the utility of health states. Results of the study showed that the WTT years and the number of years traded-off were both influenced by SLE in 10-year TTO exercises. Reducing remaining life expectancy to 10 years in a TTO may thus increase loss aversion, especially in respondents losing relatively more expected life years. These findings support the notion that our study is relevant to health economic methodology [17, 18].

In terms of age-specific association with mortality, previous study [16] indicated a positive relationship between SLE and estimated 10-year mortality risk, as well as being predictive of actual mortality risk in a national sample of older American adults. In the HRS, older adults who survived over a 2-year follow-up had a 50-point higher estimate of 15-25 year SLE than those who died [4]. In a longer 8-year follow-up, those who died reported a 56% chance of 15-25 year survival at baseline compared with a 65% average chance reported by those who survived [19].

Several potential limitations of the present study should be noted. First, data was gathered from self-reports of sociodemographic factors and health risk factors; self-report is an imperfect indicator of actual behavior. Second, our measure of life expectancy included a range of ages rather than a specific age estimate. This approach reduced the full range of the data but was more feasible for respondents. Despite our approximate measure of SLE, our findings were consistent with research obtaining precise age estimates of life expectancy [20]. With more precise age estimates, future research will need to confirm observed effects. Finally, we did not assess other factors (e.g., family medical history) that may account for the association between life expectancy and mortality.

Despite these limitations, this study has various strengths, particularly with its use of a population-based representative sample and the 8-year follow-up database. We also prospectively analyzed a large number of individuals from longitudinal data of a well-defined and comprehensively studied sample of older adults to examine the association between SLE and self-rated health, and mortality. Therefore, with the rapidly aging population in Korea, SLE is a reasonably good predictor of future mortality. Our findings leave little room for doubt about whether SLE should be taken seriously to understand future survival and to meaningfully intervene in preventing early mortality.

Inequality in mortality due to varying socioeconomic status has been shown in different countries [21, 22]. People with poor educational attainment and socioeconomic status may have lower expectations of SLE [23, 24], and this low expectation could lead to the increase in actual mortality. Therefore, further research needs to examine SLE due to different sociodemographic factors more accurately, as well as how SLE and sociodemographic factors affect actual mortality [21, 22]. There is a strong relationship between SLE and an individual’s sense of control for the future [25], and this could consequently lead to increased likelihood of participating in health promoting programs that may eventually decrease the risk of mortality.

## Conclusion

In conclusion, this study has shown that self-rated health is found to be an independent predictor of SLE and that SLE is a powerful predictor of mortality after adjusting for self-rated health as well as sociodemographic factors, health risk status and behavioral factors in Korean representative population. Further research is needed to examine the accuracy of SLE across socioeconomic groups and its predictive ability in different socioeconomic groups for mortality.

## Abbreviations

GEE: Generalized estimating equation
HR: Hazard ratio
HRS: Health and Retirement Study
KLoSA: Korean Longitudinal Study of Aging
OR: Odds ratio
SLE: Subjective life expectancy
TTO: Time trade-off
WTT: Willingness to trade-off

## References

[1] Siegel M, Bradley EH, Kasl SK (2003). Self-rated life expectancy as a predictor of mortality: evidence from the HRS and AHEAD surveys. Gerontology.

[2] Manski C (2004). Measuring expectations. Econometrica.

[3] Zimbardo PG, Boyd JN (1999). Putting time into perspective: a valid, reliable individual-difference metric. J Pers Soc Psychol.

[4] Smith VK, Taylor DH, Sloan FA (2001). Longevity expectations and death: can people predict their own demise. Am Econ R.

[5] Rappange DR, Brouwer WB, van Exel J (2016). Rational expectations? An explorative study of subjective survival probabilities and lifestyle across Europe. Health Expect.

[6] Cate RA, John OP (2007). Testing models of the structure and development of future time perspective: maintaining a focus on opportunities in middle age. Psychol Aging.

[7] Hesketh B, Griffin B (2010). Retirement planning survey 2009: NSW Department of Premier and Cabinet (Research Report P2010_015).

[8] Perozek M (2008). Using subjective expectations to forecast longevity: Do survey respondents know something we don't know?. Demography.

[9] Elder TE (2013). The predictive validity of subjective mortality expectations: evidence from the Health and Retirement Study. Demography.

[10] Jylhä M, Rogers RG, Crimmins EM (2011). Self-rated health and subjective survival probabilities as predictors of mortality. International handbooks of population 2: International handbook of adult mortality.

[11] Woo H, Zajacova A. Predictive strength of self-rated health for mortality risk among older adults in the United States: Does it differ by race and ethnicity? Res Aging. 2017;39(7):879–905. 10.1177/0164027516637410 26993957

[12] Jylha M (2009). What is self-rated health and why does it predict mortality? Towards a unified conceptual model. Soc Sci Med.

[13] Hamermesh DS (1985). Expectations, life expectancy, and economic behavior. Q J Econ.

[14] van Solinge H, Henkens K (2010). Living longer, working longer? The impact of subjective life expectancy on retirement intentions and behaviour. Eur J Pub Health.

[15] Scott-Sheldon LA, Carey MP, Vanable PA, Senn TE (2010). Subjective life expectancy and health behaviors among STD clinic patients. Am J Health Behav.

[16] Kobayashi LC, von Wagner C, Wardle J. Perceived life expectancy is associated with colorectal cancer screening in England. Ann Behav Med. 2016; E-pub 10.1007/s12160-016-9855-z PMC5440484 27822612

[17] van Nooten FE, Koolman X, Brouwer WB (2009). The influence of subjective life expectancy on health state valuations using a 10 year TTO. Health Econ.

[18] van Nooten F, Brouwer W (2004). The influence of subjective expectations about length and quality of life on time trade-off answers. Health Econ.

[19] Hurd MD (2009). Subjective probabilities in household surveys. Annu Rev Econom.

[20] Brouwer WBF, van Exel NJA (2005). Expectations regarding length and health related quality of life: Some empirical findings. Soc Sci Med.

[21] Larfors G, et al. The impact of socioeconomic factors on treatment choice and mortality in chronic myeloid leukaemia. Eur J Haematol. 2016. 10.1111/ejh.12845 28009456

[22] Teng AM, et al. Changing socioeconomic inequalities in cancer incidence and mortality: Cohort study with 54 million person-years follow-up 1981-2011. Int J Cancer. 2016. 10.1002/ijc.30555 27925183

[23] Landow S, et al. The effect of socioeconomic status, ethnicity, and sex on subjective life expectancy among adolescent Rhode Islanders. Med Health R I. 1998;81(1):7–10. 9473934

[24] Mirowsky J, Ross CE. Socioeconomic status and subjective life expectancy. Soc Psychol Q. 2000;63:133–51.

[25] Mirowsky J. Age, subjective life expectancy, and the sense of control: the horizon hypothesis. J Gerontol B Psychol Sci Soc Sci. 1997;52(3):S125–34. 10.1093/geronb/52b.3.s125 9158569

